# Brain Morphology and Quantitative Assessment of Sensory Brain Areas in Southern Bluefin Tuna, *Thunnus maccoyii* (Scombridae, Teleostei)

**DOI:** 10.1002/cne.70148

**Published:** 2026-03-27

**Authors:** Myoung Hoon Ha, Lucille Chapuis, Rebecca Glarin, Bradford Moffat, David K. Wright, Travis L. Dutka, Julian Pepperell, Caroline C. Kerr, Kara E. Yopak, Shaun P. Collin

**Affiliations:** ^1^ School of Agriculture, Biomedicine and Environment La Trobe University Melbourne Victoria Australia; ^2^ Melbourne Brain Centre Imaging Unit, Department of Radiology, Medicine, Dentistry and Health Sciences University of Melbourne Melbourne Victoria Australia; ^3^ School of Translational Medicine Monash University Melbourne Victoria Australia; ^4^ Pepperell Research & Consulting Pty Ltd Doonan Queensland Australia; ^5^ Max Planck Queensland Centre (MPQC) for the Materials Science of Extracellular Matrices Queensland University of Technology Kelvin Grove Queensland Australia; ^6^ Department of Biology and Marine Biology University of North Carolina Wilmington, UNCW Center for Marine Science Wilmington North Carolina USA

**Keywords:** corpus cerebelli, comparative brain morphology, magnetic resonance microscopy, optic tectum, pelagic teleost, specialization

## Abstract

A quantitative comparison of the absolute and relative volumes of different brain areas is useful for predicting the sensory capabilities and behavior of large pelagic teleosts, which are difficult to study in the field or in vivo. However, the size of pelagic teleost brain regions has only been approximated using the idealized ellipsoid method, which is susceptible to over‐ or underestimation, as it assumes the shape of brain regions to be an idealized ellipsoid or half‐ellipsoid. This study examines the gross morphology and volumes of different sensory brain areas of southern bluefin tuna *Thunnus maccoyii* using magnetic resonance imaging (MRI). The results show that the optic tectum (568 ± 11 mm^3^) has a larger absolute volume compared to the olfactory bulb (50 ± 5 mm^3^), eminentia granularis (62 ± 9 mm^3^), and cristae cerebelli (47 ± 3 mm^3^), suggesting the significance of vision for *T. maccoyii*. The full segmentation of a *T. maccoyii* brain allowed the quantification of the integration areas, which reveals that the corpus cerebelli (1299 mm^3^) occupies the largest proportion (35%) of total brain volume, whereas the optic tectum only occupies 15% of total brain volume. The corpus cerebelli also exhibits a rostro‐caudal elongation with multiple horizontal sulci, which resemble the corpus cerebelli of some species of sharks. The results reveal that the brain of *T. maccoyii* is dominated by the locomotive area of the corpus cerebelli and highlight the benefits of using MRI when performing quantitative analyses on the brain volumes of large pelagic teleosts.

## Graphical abstract

The absolute and relative volume of major sensory and integrative brain regions of the southern bluefin tuna *Thunnus maccoyii* are examined using magnetic resonance imaging (MRI). A dominant cerebellum projects into the midbrain ventricular space, while the optic tectum represents a smaller proportion of total brain volume than previously estimated using idealized ellipsoid methods.

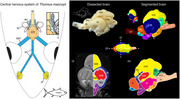

## 1 Introduction

Structural morphology of peripheral sensory organs has often been used as an indicator of ecological adaptation in fishes. Pelagic or mesopelagic fishes that rely on vision to navigate the dimly lit environments possess enlarged eyes and wide, long photoreceptors to maximize photon capture (Fernald 1988; Fritsches et al. 2003; Warrant et al. 2003; Brill et al. 2005), while benthic or demersal fishes that rely on chemoreception for feeding have distinct external structures like barbels or numerous external taste buds (Gomahr et al. 1992; Harvey and Batty 1998; Kiyohara and Tsukahara 2005; Nakamura et al. 2017). The afferent sensory nerves that connect the peripheral sense organs to the central (sensory) brain areas also reflect the relative importance of each sensory modality, not only in teleosts but in vertebrates more broadly (Köppl 1997; Leitch et al. 2014; Wohlert et al. 2016; Lisney et al. 2018). For instance, barn owls (*Tyto alba*), auditory specialists, have a higher number of axons in their auditory nerve compared to other avian species (Köppl 1997). The axon numbers in the olfactory tracts of deep‐sea grenadiers (e.g., *Coryphaenoides armatus*, *C. profundicolus*) increase while the axon number in their optic nerves decreases as they mature and adopt a more scavenging lifestyle (Lisney et al. 2018). These peripheral specializations are mirrored in the central nervous system (CNS), which functions as a control center for integrating sensory input and generating behavioral responses and reflects an animal's lifestyle (Nieuwenhuys et al. 1998). Distinct brain regions process afferent input from different sensory modalities (Butler 2011). For example, the olfactory bulb is the first‐order processing center for olfactory input (Finger 1988; Camilieri‐Asch et al. 2020), the optic tectum processes the majority of visual input (Northcutt and Wullimann 1988), and the octavolateral areas process auditory and vestibular information (McCormick and Braford 1988). Among vertebrates, these individual brain regions vary in relative size, likely due to a combination of different selection pressures (Liao et al. 2015; Yopak et al. 2015; Kotrschal et al. 2017), coupled with environmental (Barton et al. 1995; Gutierrez‐Ibanez et al. 2013; Yamamoto, 2017; Iglesias et al. 2018) and ontogeny (Wagner 2003; Lisney et al. 2007). Variation in the volume of each brain region, in turn, is assumed to be at least partially due to the “principle of proper mass” (Jerison 1973). This principle states that the relative size of a brain region should reflect the relative importance of the function of that brain region.

Teleosts, comprising more than 36,000 species (Fricke et al. 2025), present extensive cases for investigating how sensory functions required by habitat and behavioral conditions influence brain morphology. It has been widely observed that the dominant sensory modality in a given teleost species is associated with a disproportionately larger corresponding brain region (Kotrschal et al. 1998; Yamamoto 2017; Axelrod et al. 2021). Visually‐oriented pelagic teleosts (e.g., tunas and billfishes) or diurnal teleosts occupying a bright, visually complex habitat like coral reefs exhibit a relatively larger optic tectum compared to their olfactory bulb or octavolateralis areas, suggesting a greater investment in vision and visual processing (Burr 1928; Kawamura et al. 1981; Lisney and Collin 2006; Palmieri et al. 2008; Iglesias et al. 2018). Teleosts that hunt in low‐light conditions (i.e., nocturnal or deep‐sea) often show reduced optic tectum volume (Yamamoto 2017; Iglesias et al. 2018), suggesting a reduction in the relative importance of vision in scotopic environments. Some piscivores, instead, may specialize more in olfaction and correspondingly present an increase in the size of the olfactory bulb (Wagner 2003; Edmunds et al. 2016). This relationship between regional specialization and size is not limited to brain regions that receive direct afferents from peripheral sensory systems. Integration centers, such as the telencephalon and corpus cerebelli, also correlate with ecological and behavioral parameters (Axelrod et al. 2021). For example, large, fast‐swimming pelagic teleosts tend to have a relatively enlarged corpus cerebelli (Lisney and Collin 2006), which supports complex motor coordination and motor learning (Llinas 1985), although its exact functionality is still in contention (Paulin 1993). Teleosts that exhibit intricate social behaviors, such as dominance hierarchies or courtship behaviors, often possess a well‐developed telencephalon (Kotrschal et al. 1998; Lisney and Collin 2006).

Volumetric analyses of brain regions may therefore provide valuable insights into the sensory or behavioral specializations of animals and are especially useful for studying species that are difficult to access or observe in the wild, such as large pelagic teleosts. Despite this potential, the brains of pelagic teleosts have been quantified in only a few studies (e.g., Wagner 2001; Lisney and Collin 2006), and existing descriptions are often limited to qualitative observations based on gross morphology (Burr, 1928; Kawamura et al. 1981; Palmieri et al. 2008). With respect to tunas (Scombridae: Thunnini), one of the most commercially important groups of fishes globally, major brain structures have been quantitatively analyzed in only one species, the skipjack tuna *Katsuwonus pelamis* (Lisney and Collin 2006). No quantitative data currently exist on sensory or integrative brain regions of any species within the genus *Thunnus*, which includes the iconic bluefin tunas. As is common in comparative neuroanatomical studies in teleosts (e.g., Wagner 2001, 2002, 2003; Lisney and Collin 2006), the volume of the different brain areas of *K. pelamis* was measured using the “idealized ellipsoid method” (Lisney and Collin 2006), which assumes that each brain area approximates the volume of an idealized ellipsoid or half‐ellipsoid (Huber et al. 1997). The presence of a well‐developed optic tectum, in addition to large eyes, has been a key reason why tunas, including *K. pelamis*, are widely regarded as visually‐oriented animals (Tamura and Wisby 1963; Kawamura et al. 1981; Lisney and Collin 2006).

The ellipsoid method is the most efficient method of estimating the relative size of different brain regions, as it does not require histological processing and sectioning (Ullmann et al. 2011; Yopak and Lisney 2012; Salas et al. 2015). However, the ellipsoid method assumes the shape of the brain area to be ellipsoid or half‐ellipsoid, which can over‐ or underestimate the volume of sensory brain tissue based on the inclusion of ventricular spaces and/or the inability to differentiate sensory and non‐sensory tissue (Ullmann et al. 2011). Apart from the olfactory bulbs, most major brain regions in fishes do not perfectly fit into an ellipsoid or a half‐ellipsoid (Ullmann et al. 2011; Salas et al. 2017). The optic tectum, which appears to be a dominant element of the midbrain when viewed externally, is a multilayered structure that comprises the roof of the midbrain, with other midbrain regions assigned to nonvisual functions (Eastman and Lannoo 2004; Cerdá‐Reverter et al. 2008; Ullmann, Cowin, and Collin 2010; Ullmann et al. 2011). Hence, using the ellipsoid method to measure the volume of the optic tectum in teleosts can lead to significant overestimation of tissue volume dedicated to visual processing. Indeed, the volume of the optic tectum of the barramundi *Lates calcarifer*, calculated using the ellipsoid method, was revealed to be 250% greater than that obtained using magnetic resonance imaging (MRI) (Ullmann et al. 2011).

To address this, quantitative analyses of brain structures using advanced imaging techniques are needed, particularly to overcome the limitations of traditional ellipsoid‐based volume estimation methods. MRI has become increasingly popular in comparative neuroanatomy due to its ability to visualize both peripheral and central brain structures at high resolution (e.g., Yopak and Frank 2009; Ullmann, Cowin, Kurniawan, et al. 2010; Ullmann, Cowin, and Collin 2010; Ullmann et al. 2011; Yopak et al., 2019; Collin et al. 2024). Virtual segmentation of MRI datasets in any three‐dimensional (3D) plane allows for precise volumetric analysis, without the risk of under‐ or overestimation of absolute or relative brain volumes (Ullmann et al. 2011). Therefore, MRI‐based examination of brain morphology in pelagic teleosts can be a critical tool to accurately assess how their neural architecture reflects a pelagic lifestyle.

The aims of this study are to examine the morphology of the brain and quantitatively assess the volume of sensory brain areas in the southern bluefin tuna (SBT) *Thunnus maccoyii* (Teleostei), using MRI. Regions of interest (ROIs) include the sensory areas of the olfactory bulbs, optic tectum, and octavolateralis region (comprising the eminentia granularis and cristae cerebelli), and the integrative areas, including the telencephalon, corpus cerebelli, and valvula cerebelli.

## 2 Materials and Methods

### 2.1 Source of Animals and Brain Preservation

The four heads of SBT (ranging from 95 to 101 cm in total length [L_T_]) used in this study were donated by recreational fishermen under Victorian Fisheries Authority research permit #1447. The SBT were all line caught in the central Bass Strait, Victoria, Australia. Upon capture, all specimens were decapitated, and the heads were immersed in 10 L of 4% paraformaldehyde (PFA) in 0.1 M phosphate buffer (PB) that was chilled at approximately 4°C to optimize fixation of the brain and neurocranium.

### 2.2 Magnetic Resonance Imaging Acquisition

Two SBT heads (SBT8 and SBT9) fixed in 4% paraformaldehyde (PFA) in 0.1M PB were immersed in 10 L of PB azide solution (0.1 M, 0.01%) for 3 weeks to rinse out residual fixative. Following this, 8 L of fresh PB azide solution (0.1 M, 0.01%) was prepared, and 43.76 g of diethylenetriaminepentaacetic acid gadolinium (III) dihydrogen salt was added to achieve a final concentration of 10 mM/L of gadolinium salt (the gadolinium salt solution). The heads were then immersed in the gadolinium solution for 8 weeks to enhance the contrast of the brain structures for MRI. To facilitate contrast agent diffusion and reduce the overall volume, the snout and eyes were removed from each head prior to immersion. MRI data were acquired in situ on a Siemens Magnetom 7T Plus system (Germany), in a 1Tx/32Rx Head coil (Nova Medical) using a turbo FLASH sequence (Haase et al. 1986) at The University of Melbourne. The 3D MRI data consisted of 0.2 mm isotropic voxels with a 200 mm × 200 mm in‐plane field of view and 192 slices (100% slice oversampling) acquired with 7/8 partial Fourier coverage in the slice and phase encode directions. The repetition time (TR) was 13 ms with an echo time (TE) of 5 ms. To improve the signal‐to‐noise ratio, four averages were acquired, resulting in a total imaging time of 2 h and 14 mins. The images were then analyzed using Dragonfly (Comet Technologies Canada Inc., version 2024).

The brains from a further two individuals (SBT16 and SBT18) were carefully dissected from their neurocrania, both previously immersion fixed in 4% PFA in 0.1 M PB and prepared for separate MRI sessions. The same protocol was followed, involving initial immersion in PB azide solution (0.1 M, 0.01%) to remove residual fixatives, followed by immersion in the gadolinium‐enhanced solution. However, since the brains were dissected prior to immersion, rather than processed as whole heads, the brains were immersed and monitored to achieve optimal contrast and reduce exposure time to prevent overstaining: 1 week in PB azide (reduced from 3 weeks), and 1 week in the gadolinium solution (reduced from 8 weeks). Images of the two excised brains were acquired on a Bruker (Germany) 9.4T MRI scanner at Monash University, Melbourne, Australia. Acquisition parameters were as follows: TR = 40 ms, TEs = 4.5, 12, 19.5 ms, pulse (flip) angle = 90°, field‐of‐view = 28.8 × 21.6 × 21.6 mm^3^, acquisition matrix = 384 × 288 × 288, giving an image with an isotropic resolution of 75 µm. Four averages were acquired, resulting in a total imaging time of 3 h and 41 mins. The acquired 3D images were imported and analyzed using Dragonfly (Comet Technologies Canada Inc., version 2024).

### 2.3 Segmentation and Quantification of Brain Regions

Manual segmentation was performed using Dragonfly (Comet Technologies Canada Inc., version 2024). For each specimen, the following sensory brain areas were segmented, and their volumes quantified: olfactory bulb, telencephalon, optic tectum, torus longitudinalis, eminentia granularis, and cristae cerebelli. The brain of specimen SBT8 exhibited superior preservation and image contrast compared with the others, enabling additional segmentation and volume quantification of the inferior lobe, hypophysis, corpus cerebelli, and valvula cerebelli. Neuroanatomical structures within the brain were identified based on location, shape, and grayscale intensity. As no brain atlas currently exists for *T. maccoyii*, boundaries of sensory brain regions were determined with reference to multiple anatomical sources (McCormick and Braford 1988; Butler and Northcutt 1993; Cerdá‐Reverter et al. 2001, 2008; Palmieri et al. 2008; Ullmann, Cowin, Kurniawan, et al. 2010; Ullmann, Cowin, and Collin 2010). Segmentation was conducted manually, slice by slice, in all three planes (horizontal, axial, and sagittal), to verify the boundaries of major brain structures. Only regions with clearly defined anatomical boundaries were segmented; structures with ambiguous borders were either grouped or outlined with dashed lines. Upon completion, the absolute volume (mm^3^) of each segmented brain structure was automatically calculated. A standardized color‐coded scheme was applied to facilitate clear differentiation of brain regions in both 3D surface renderings and two‐dimensional (2D) section views.

## 3 Results

### 3.1 Central Nervous System of *T. maccoyii*

**FIGURE 1:**
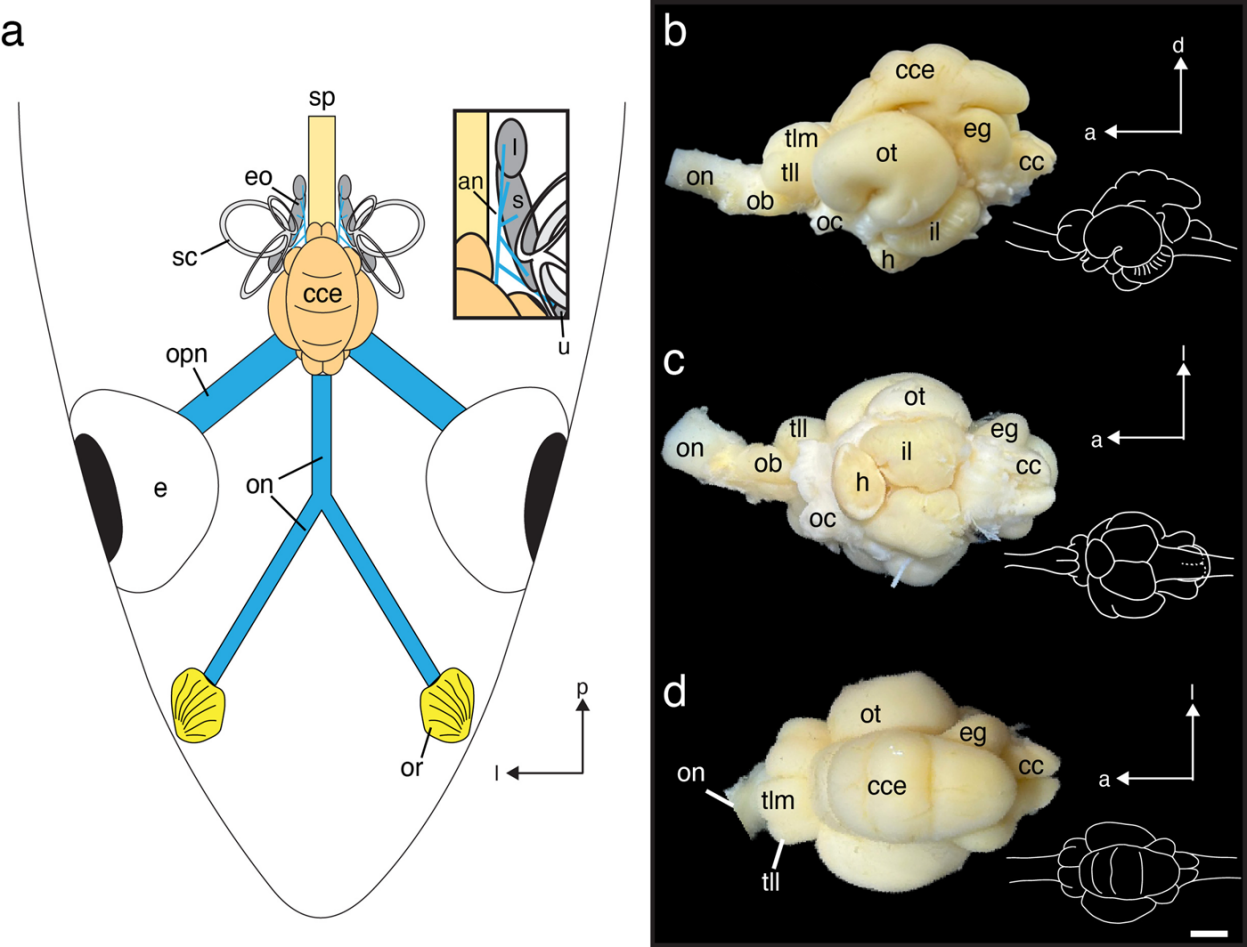
(a) Illustration of the peripheral and central nervous system of *Thunnus maccoyii* from a dorsal view (not to scale). Inset shows the magnified view of the inner ear end organs and auditory nerves. (b–d) Photographs of the brain of *T. maccoyii*. Drawings on the right show the missing spinal cord and posterior portion of the medulla. (b) Lateral aspect. (c) Ventral aspect. (d) Dorsal aspect. Note the deep grooves on the corpus cerebelli. Orientation: a, anterior; d, dorsal; l, lateral; p, posterior. an, auditory nerve; cc, cristae cerebelli; cce, corpus cerebelli; e, eye; eg, eminentia granularis; eo, end organ; h, hypophysis; il, inferior lobe; l, lagena; m, medulla; ob, olfactory bulb; oc, optic chiasma; on, olfactory nerve; opn, optic nerve; or, olfactory rosette; ot, optic tectum; s, saccule; sc, semicircular canal; sp, spinal cord; tll, telencephalon lateral lobe; tlm, telencephalon medial lobe; u, utricle. Scale bars, 5 mm (b–d).

**FIGURE 2:**
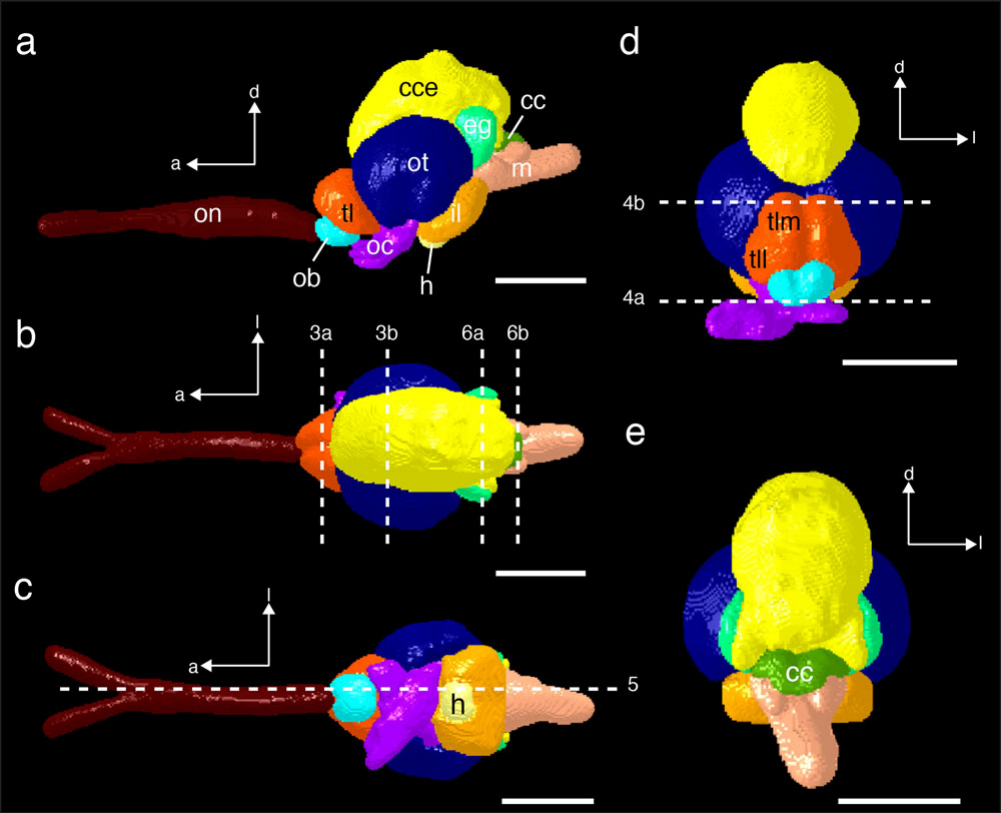
Digital segmentation of the major structures of the brain of *T. maccoyii* (SBT8) using Dragonfly (Comet Technologies Canada Inc, version 2024) in (a) lateral, (b) dorsal, (c) ventral, (d) frontal, and (e) caudal views. (a) Major neuronal structures labelled. (b) Dashed lines indicate levels of the axial sections used in Figures 3 and 6. (c) Dashed line indicates the level of the sagittal section used in Figure 5. (d) Dashed lines indicate levels of the horizontal sections used in Figure 4. Orientation: a, anterior; d, dorsal; l, lateral. cc, cristae cerebelli; cce, corpus cerebelli; eg, eminentia granularis; h, hypophysis; il, inferior lobe; m, medulla; ob, olfactory bulb; oc, optic chiasma; on, olfactory nerve; ot, optic tectum; tl, telencephalon; tll, telencephalon lateral lobe; tlm, telencephalon medial lobe. Scale bars, 10 mm (a–c); 8 mm (d, e).

The brain in *T. maccoyii* is positioned along the midline, with the forebrain aligned with the caudal end of the eyes (Figure 1a). The brain is protected within a thick neurocranium, and its dorsal and lateral surfaces are covered by a thick dura with fat deposits. The long olfactory nerves arise from the peripheral olfactory rosettes and terminate in sessile olfactory bulbs, which are distant from the olfactory rosettes and proximal to the telencephalon. Interestingly, the left and right olfactory nerves converge into a single nerve bundle, which is as thick as the width of the olfactory bulb, and terminates within the olfactory bulb (Figures 1 and 2). The olfactory bulb consists of a pair of lobes attached ventrally to the telencephalon, which extends rostrally. These lobes represent the most rostral part of the brain (Figure 1b–d). The olfactory bulbs are not visible from the dorsal aspect (Figure 1d) due to the thickness of the olfactory nerve (about 1.87 mm in diameter) and a relatively large telencephalon. The short olfactory tracts connecting the olfactory bulbs to the telencephalon are not visible externally. The telencephalon can be externally differentiated into a pair of medial and lateral lobes (Figure 1d). Immediately caudal to the telencephalon is the prominent optic tectum, which is a pair of large lobes with deep grooves (sulci) on their ventrolateral surfaces. Qualitatively, the optic tectum dominates the midbrain (Figure 1b) and is connected to the eyes via large optic nerves (about 2.8 mm in diameter), which project contralaterally via the optic chiasm, which is best observed ventrally (Figure 1b,c). Ventral to the optic tecta are a pair of prominent inferior lobes and a hypophysis or pituitary gland, which projects rostrally (Figure 1d). Dorsal to the optic tectum is the rostro‐caudally elongated corpus cerebelli, which extends from the telencephalon to the eminentia granularis (Figure 1b). Three horizontal sulci can be found on the dorsal surface of the corpus cerebelli (Figure 1b,d). Ventral to the caudal portion of the corpus cerebelli are the paired lobes of the eminentia granularis, which show distinct protuberances (Figure 1b–d). The cristae cerebelli can be found caudal to the eminentia granularis and dorsal to the remainder of the medulla oblongata, which receives projections from the auditory component of the VIIIth cranial nerves arising from the three otolithic end organs of the inner ears (saccule, lagena, and utricle) (Figure 1a). The facial and vagal lobes are not distinct and cannot be differentiated from other regions of the hindbrain. The spinal cord is connected to the caudal end of the medulla oblongata and extends caudally.

### 3.2 Brain Morphology of *T. maccoyii*

The MRI scans and subsequent segmentations reveal the internal brain structures, their orientation, and regional interrelationships in situ (Figure 2a–e; Supporting Information S1–S4). The following sections describe the internal structures of the main brain divisions in *T. maccoyii*.

### 3.3 Forebrain

**FIGURE 3:**
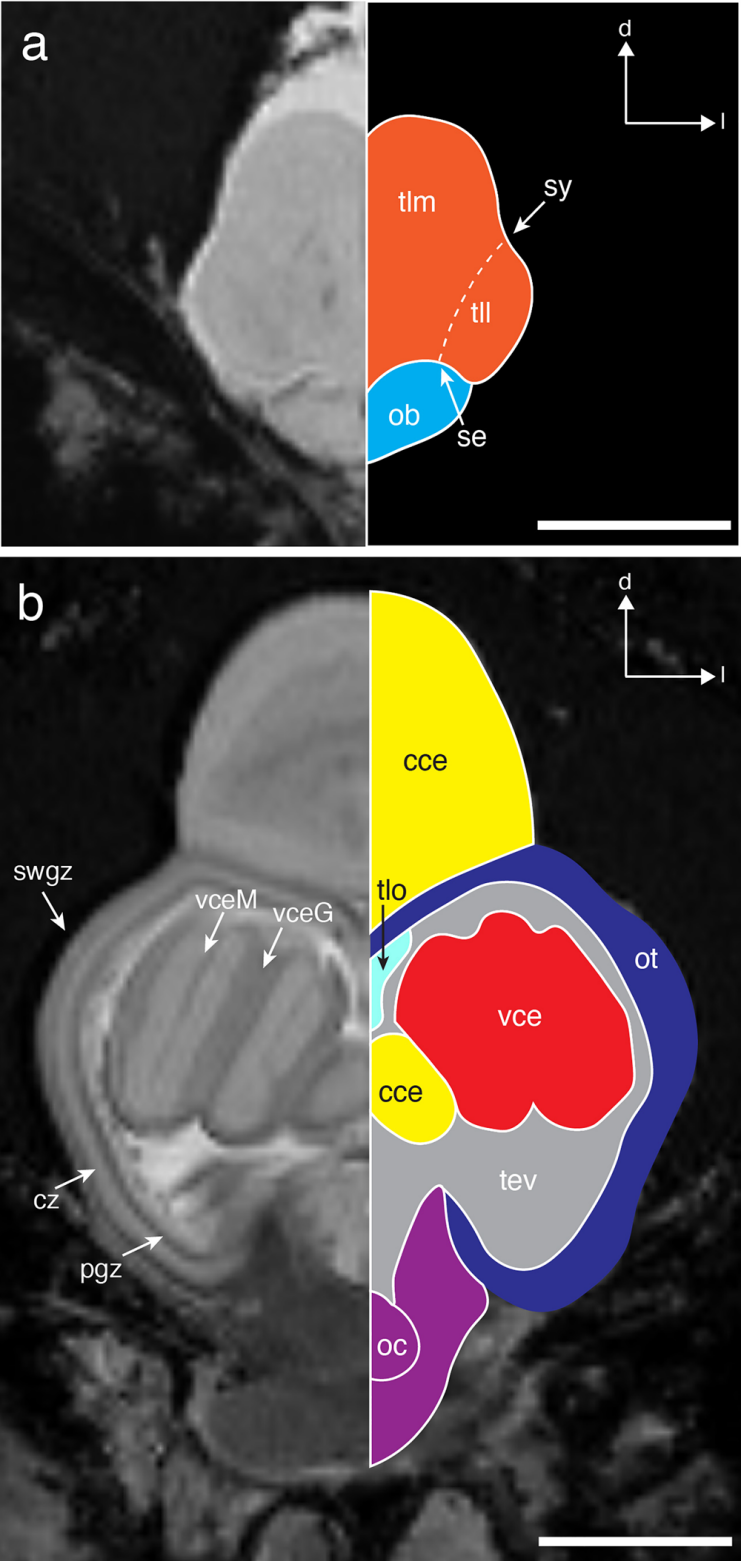
A pair of axial slices through the forebrain (a) and midbrain (b) of *T. maccoyii*. MRI 2D slices are on the left, and the line tracings on the right indicate different regions. Each region is colored with colors corresponding to the 3D segmentations, while the ventricle and areas that cannot be differentiated are rendered in gray). Dashed line indicates a likely boundary. Note that the optic tectum only comprises the roof of the midbrain. Orientation: d, dorsal; l, lateral. cc, corpus cerebelli; cs, connective sheath; cz, central zone of the ot; ob, olfactory bulb; oc, optic chiasma; ot, optic tectum; pgz, periventricular gray zone of the ot; se, sulcus externus; swgz, superficial gray and white zone of the ot; sy, sulcus ipsilyformis; tev, tectal ventricle; tll, telencephalon lateral lobe; tlm, telencephalon medial lobe; tlo, longitudinal torus; vce, valvula of cerebelli; vceG, granular layer of the vce; vceM, molecular layer of the vce. Scale bars, 5 mm (a, b).

**FIGURE 4:**
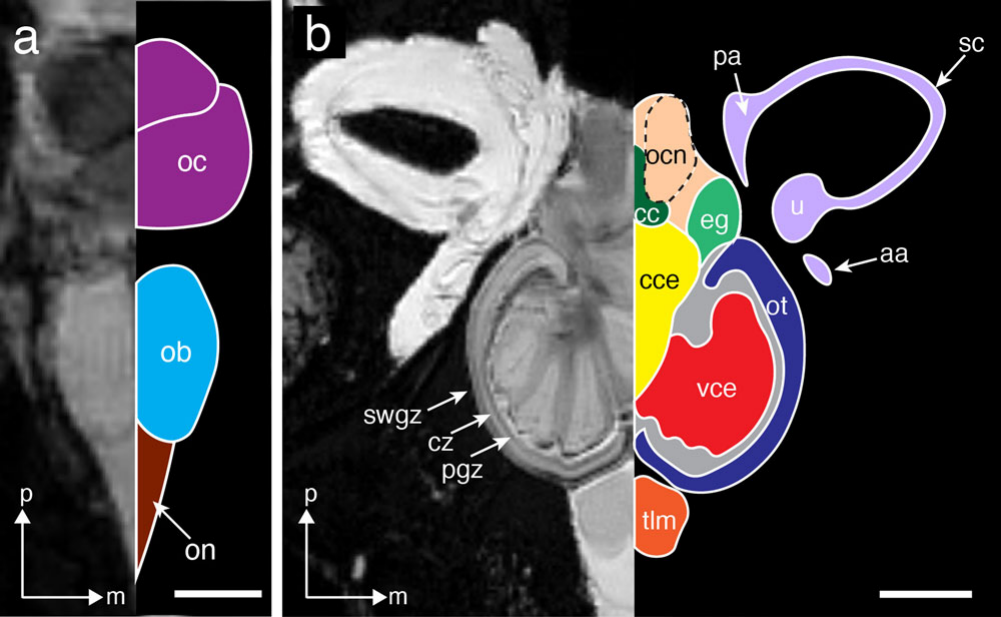
(a) Transverse MRI 2D slice through the olfactory bulb. The crossing of the optic nerves at the chiasm is also visible. (b) Mid‐transverse MRI 2D slice. The MRI image is on the left, and line tracings on the right indicate different regions. Each region is colored with colors corresponding to the 3D segmentations, while the ventricle and areas that cannot be differentiated are rendered in gray. Inner ear structures are colored with light purple. Dashed line indicates the likely boundary. The membranous structures of the inner ear are also shown adjacent to the brain. Orientation: m, medial; p, posterior. aa, anterior ampulla; cc, cristae cerebelli; cce, corpus cerebelli; cz, central zone of the ot; eg, eminentia granularis; ocn, octavolateral nucleus; ot, optic tectum; pa, posterior ampulla; pgz, periventricular gray zone of the ot; sc, semicircular canal; swgz, superficial gray and white zone of the ot; tlm, telencephalon medial lobe; u, utricle; vce, valvula of cerebelli. Scale bars, 2.5 mm (a); 5 mm (b).

**FIGURE 5:**
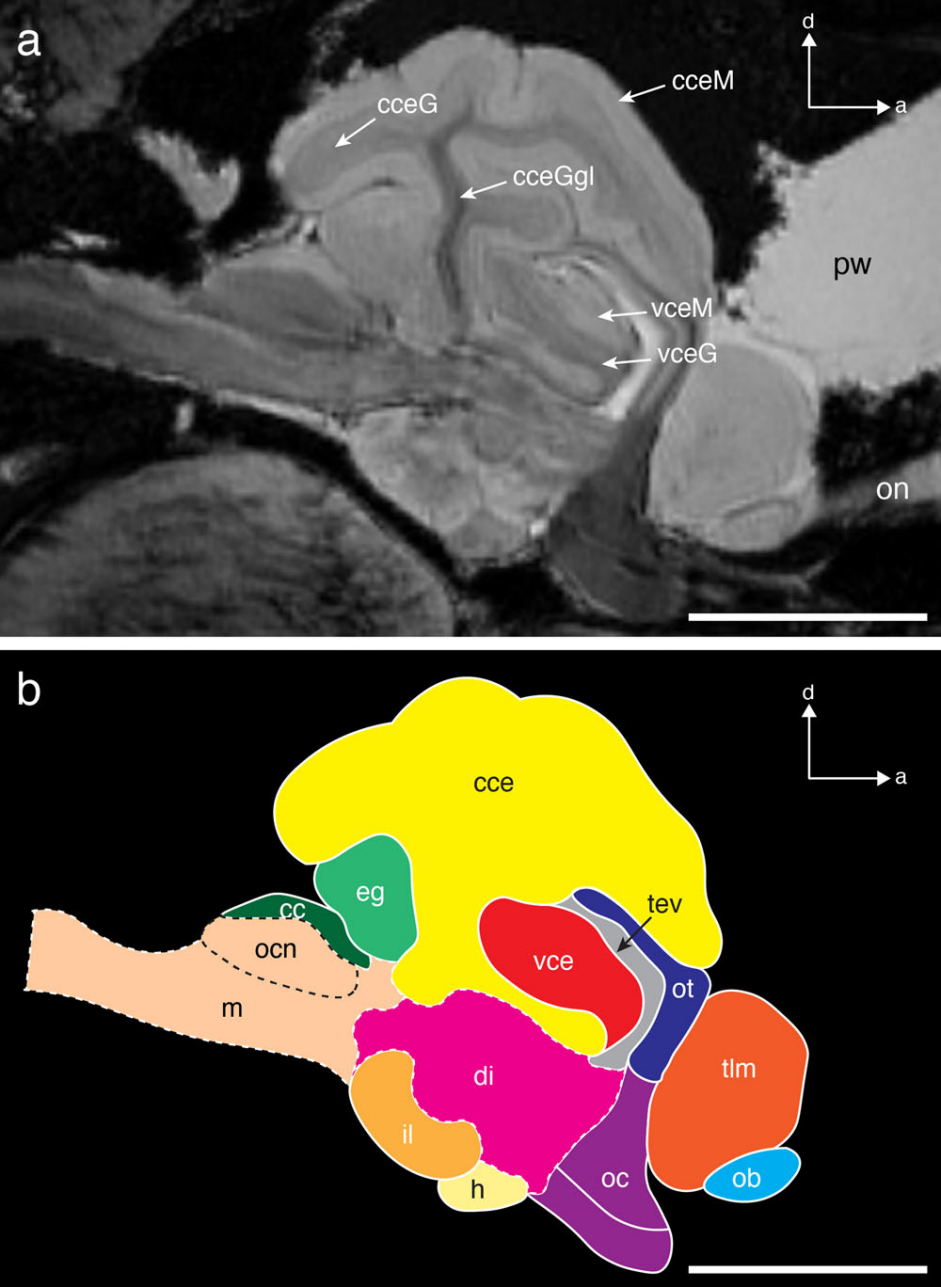
Mid‐sagittal MRI image (a) and corresponding line tracing (b) of the brain of *T. maccoyii*. Each region is colored with colors corresponding to the 3D segmentations, while the ventricle is rendered in gray. Dashed lines indicate likely boundaries. Orientation: a, anterior; d, dorsal. cc, cristae cerebelli; cce, corpus cerebelli; cceG, granular layer of the cce; cceGgl, ganglion layer of cerebellum; cceM, molecular layer of the cce; cs, connective sheath; di, diencephalon; eg, eminentia granularis; h, hypophysis; il, inferior lobe; m, medulla; ob, olfactory bulb; oc, optic chiasma; ocn, octavolateral nucleus; on, olfactory nerve; ot, optic tectum; pw, pineal window; tev, tectal ventricle; tlm, telencephalon medial lobe; vce, valvula of cerebelli; vceG, granular layer of the vce; vceM, molecular layer of the vce. Scale bars, 10 mm (a, b).

The forebrain comprises the olfactory bulb, telencephalon, and diencephalon. The paired olfactory bulbs (ob) are attached ventrally to the telencephalon (tl) and are rostrally connected to the olfactory nerve, which comprises the two lateral nerves bound by a single connective sheath (Figures 3a and 4a). The telencephalon is divided into left and right lobes along the mid‐sagittal plane, with each lobe differentiated into a medial (tlm) and lateral lobe (tll) at the sulcus ipsilyformis (sy) dorsally, and the sulcus externus (se) ventrally (Figure 3a). The resolution of the MRI images (200 × 200 × 200 µm^3^/voxel) did not allow further subdivision of the internal structure of the telencephalon. The rostro‐dorsal part of the telencephalon leads to the prominent cartilaginous pineal window, a tube‐like structure that extends all the way to the surface of the head and appears as a distinct white oval spot on the surface of the head (Figure 5a). The diencephalon is located ventral to the optic tectum and caudal to the telencephalon (Figure 5b). Among various subdivisions of the diencephalon, only the inferior lobes and the pituitary gland (or hypophysis) of the hypothalamus can be differentiated (Figure 5b). The inferior lobes are paired structures, and the pituitary gland appears rostrally. The optic chiasm appears much darker than the rest of the brain (Figures 3b, 4a, and 5b) and crosses the midline to terminate in the optic tectum ventrally (Figure 5b).

### 3.4 Midbrain

The midbrain comprises the optic tectum (ot), torus longitudinalis (tlo), tegmentum, and tectal ventricle (tev) (Figures 3b, 4b, and 5b). All three primary layers of the optic tectum: the external superficial gray and white zone (swgz), the intermediate central zone (cz), and the inner periventricular gray zone (pgz) can be differentiated, and the clear division between the hypointense (i.e., dark) PGZ and the hyperintense (i.e., bright) tectal ventricle (tev) allows the delineation of the optic tectum from the internal areas of the midbrain. The optic nerves converge at the optic chiasm, crossing contralaterally, with no obvious ipsilateral projections, to enter the optic tecta ventrally (Figures 3b and 5b). Medially, a pair of longitudinal tori can be found directly below the optic tectum (Figure 3b), which runs the entire length of the optic tectum and separates caudally. The tegmentum, another major component of the midbrain, could not be further differentiated due to the MRI scan resolution.

### 3.5 Hindbrain

**FIGURE 6:**
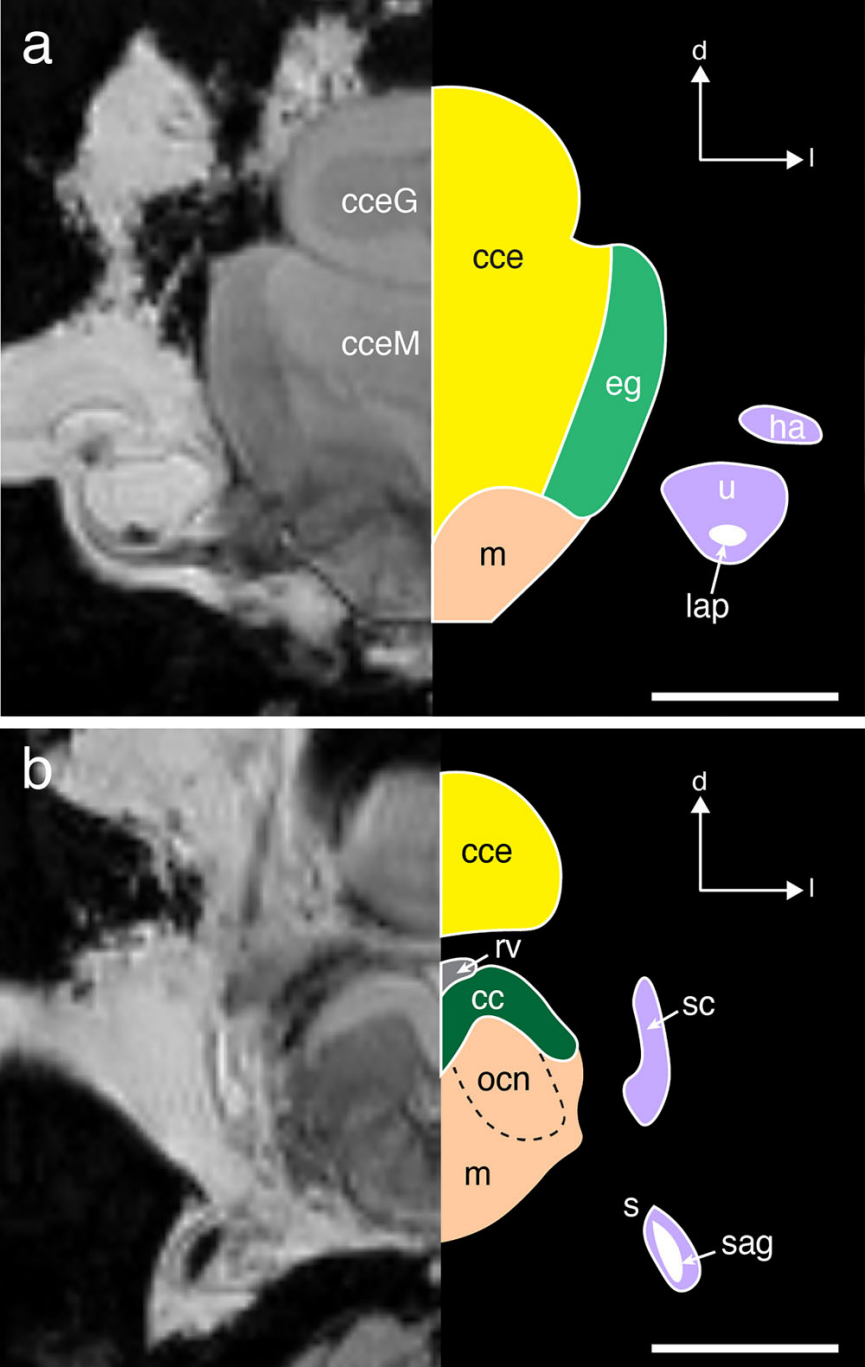
A pair of axial sections through the anterior part (a) and the posterior part (b) of hindbrain of *T. maccoyii*. The MRI image is on the left, and line tracings on the right indicate different regions. Each region is colored with colors corresponding to the 3D segmentations, while the ventricle is rendered in gray. Inner ear structures are colored with light purple. Dashed line indicates the likely boundary. The membranous structures of the inner ear: horizontal ampulla (ha), semicircular canal (sc), saccule (s), and utricle (u), and the calcified otoliths: sagitta (sag) and lapillus (lap) are also visible laterally adjacent to the brain. Orientation: d, dorsal; l, lateral. cc, cristae cerebelli; cce, corpus cerebelli; cceG, granular layer of the cce; cceM, molecular layer of the cce; eg, eminentia granularis; m, medulla; ocn, octavolateral nucleus; rv, rhombencephalic ventricle. Scale bars, 5 mm (a, b).

The hindbrain comprises the cerebellum and medulla oblongata. Two of the major structures of the cerebellum are the corpus cerebelli and valvula cerebelli, both of which protrude into the midbrain ventricle rostro‐ventrally (Figure 5b). The paired valvulae cerebelli occupy the majority of the central ventricular space of each hemisphere of the midbrain, narrowly separated from the optic tecta (Figures 3b and 4b). The valvulae cerebelli are folded into dark granular (vceG) and light molecular (vceM) layers, which can be easily differentiated (Figures 3b and 5a). The rostro‐ventral projections of the corpus cerebelli can be found along the midline of the midbrain and are contiguous with the valvula cerebelli on each side (Figures 3b and 4b), which extend anteriorly (Figure 4b). The portion of the corpus cerebelli that is dorsal to the optic tectum extends rostro‐caudally, running the entire length of the midbrain, and terminates posterior to the eminentia granularis (Figure 5a,b). There are three deep grooves (sulci) over the dorsal surface of the corpus cerebelli, beneath which lie the dark granular (cceG) and light molecular (cceM) layers (Figure 5a). The darkest layer of the corpus cerebelli, the ganglionic layer (cceGgl), can be seen more easily in the sagittal plane (Figure 5a). The third major component of the cerebellum, the eminentia granularis, extends laterally from the caudal end of the corpus cerebelli to form a distinct protuberance (Figure 6a). The paired eminentia granularis are hypointense and rostrally contiguous with the granular layer of the corpus cerebelli (Figure 6a).

The cristae cerebelli (cc), a molecular layer that covers the octavolateralis nuclei, is a region of the medulla oblongata that is also part of the octavolateralis area of the brain, receiving input from the mechanosensory (auditory and vestibular) systems. The paired cristae cerebelli comprises the dorso‐rostral surface of the medulla oblongata and are positioned ventro‐caudally to the corpus cerebelli and the eminentia granularis (Figure 6b). The cristae cerebelli can be differentiated from the rest of the medulla oblongata based on their distinct shape (wing‐like), which appears hyperintense using MRI (Figure 6b). While the resolution did not allow any further subdivision of structures within the medulla oblongata, a pair of hypointense nuclei is identified beneath the “wings” of the cristae cerebelli, which may putatively represent the octavolateralis nuclei based on their relative position (Figure 6b).

### 3.6 Quantitative Assessment of Brain Regions

Absolute and relative volumes of five sensory brain areas (olfactory bulb, optic tectum, torus longitudinalis, eminentia granularis, and cristae cerebelli) from four *T. maccoyii* were calculated following the 3D segmentations (Table 1). The mean absolute volumes of the optic tectum and torus longitudinalis, which are the primary visual targets, were 568 ± 11 mm^3^ (mean ± SE, *n* = 3) and 7 ± 1 mm^3^ (*n* = 4), respectively. The olfactory bulb has a mean volume of 50 ± 5 mm^3^ (*n* = 3). The eminentia granularis and the cristae cerebelli, which are the primary octavolateral targets, have a mean volume of 62 ± 9 mm^3^ (*n* = 3) and 47 ± 3 mm^3^ (*n* = 4), respectively.

**TABLE 1:** Quantitative assessment of the absolute volume (mm^3^) of the olfactory bulb, optic tectum, torus longitudinalis, eminentia granularis, and cristae cerebelli of the brains of *Thunnus maccoyii*.

	L_T_ (cm)	Olfactory bulb (mm^3^)	Optic tectum (mm^3^)	Torus longitudinalis (mm^3^)	Eminentia granularis (mm^3^)	Cristae cerebelli (mm^3^)
SBT8	95	42	558	6	90	42
SBT9	100	48	—	5	54	40
SBT16	101	59	556	10	51	54
SBT18	100	—	589	9	—	52
Average ± SE	99 ± 1	50 ± 5	568 ± 11	7 ± 1	62 ± 9	47 ± 3

Abbreviation: L_T_, total length.

Further segmentation of the brain of SBT8 allowed the comparison between the volume of the five sensory areas and other prominent brain structures, including the telencephalon and the cerebellum, with total brain volume (Table 2). The total volume of the SBT8 brain is 3748 mm^3^, and the five regions that include the primary olfactory, visual, and octavolateralis brain areas only comprise 19.41% of total brain volume. The optic tectum (558 mm^3^) and torus longitudinalis (6 mm^3^) comprise 15% and 0.1% of total brain volume, respectively, and the olfactory bulb (42 mm^3^) contributes 1% of the total brain volume. The eminentia granularis (80 mm^3^) and cristae cerebelli (42 mm^3^) contribute 2% and 1% to the total brain volume, respectively. In comparison, the corpus cerebelli (1299 mm^3^) is the most dominant structure, which comprises 35% of the total brain volume. Together with the valvula cerebelli (410 mm^3^), the cerebellum, without the eminentia granularis, comprises 45% of the total brain volume.

**TABLE 2:** List of color‐coded brain structures and their absolute (mm^3^) and relative volumes (%) in a *T. maccoyii* (SBT8).

SBT8	Brain region	Color	Volume (mm^3^)	Relative volume (%)
Telencephalon	Olfactory bulb		42	1
	Telencephalon		258	7
Mesencephalon	Optic tectum		558	15
	Torus longitudinalis		6	0.1
	Tectum ventricle		84	2
Diencephalon	Inferior lobe		352	9
	Hypophysis		22	0.6
Cerebellum	Corpus cerebelli		1299	35
	Valvula cerebelli		410	11
Rhombencephalon	Eminentia granularis		80	2
	Cristae cerebelli		42	1
Unlabeled	Mesen/diencephalon		309	8
	Rhombencephalon		286	8
	Total volume		3748	100

Separate from sensory brain regions, the telencephalon (258 mm^3^) comprises 7% of the total volume and is much larger in absolute size than all of the quantified sensory brain areas except for the optic tectum, which is more than twice the size of the telencephalon. The inferior lobes (352 mm^3^) are also well developed, comprising 9% of the total volume, which are larger than the telencephalon but smaller than the optic tectum or valvula cerebelli.

## 4 Discussion

Linking disproportionately enlarged brain regions to dominant sensory modalities, social behaviors, locomotion, and habitat has long been a central approach in comparative neuroanatomy (Bauchot et al. 1989; Huber et al. 1997; Kotrschal et al. 1998; Wagner 2001; Lisney and Collin 2006; Iglesias et al. 2018). Among tunas and other large pelagic teleosts, the designation of “visual predators” or “visually‐oriented animals” is primarily based on the consistent observation of a disproportionately large optic tectum in comparison to other sensory brain areas (Tamura and Wisby 1963; Kawamura et al. 1981; Lisney and Collin 2006; Palmieri et al. 2008). In this study, we quantified the volumes of major brain regions in *T. maccoyii* using MRI. To our knowledge, this represents the first application of MRI‐based volumetric analysis in a large pelagic teleost.

The external morphology of the brain of *T. maccoyii* appears similar to that of previously studied pelagic teleosts, with prominent optic tecta, a relatively well‐developed hindbrain, telencephalon, corpus cerebelli, and relatively small olfactory bulbs (Kawamura et al. 1981; Lisney and Collin 2006; Palmieri et al. 2008). However, the use of MRI in this study enabled more detailed investigation and quantification of other central structures of the midbrain in *T. maccoyii*, notably allowing nonvisual regions to be excluded from tectal measurements during segmentation. This revealed that the optic tectum is not as dominant as external observations alone would suggest, particularly when considered in relation to total brain volume, likely due to an inability to exclude the tectal ventricle when using the ellipsoid method to estimate brain region volumes (Ullmann et al. 2011).

### 4.1 Optic Tectum of *T. maccoyii*

The optic tectum of *T. maccoyii*, which can be delineated by its three distinct layers (superficial gray and white zone, central zone, and periventricular gray zone), comprises the roof of the midbrain, while the ventricular space of the midbrain, or tectal ventricle, is occupied by the valvula cerebelli and a rostral‐ventral projections of the corpus cerebelli (Supplementary Information S5). The optic tectum of *T. maccoyii*, which receives input from the retinal ganglion cells within the eye, occupies approximately 15% of the total brain volume. This is a much larger proportion of tissue than other sensory brain areas in this species (olfactory bulb, eminentia granularis, and cristae cerebelli) that receive input from the peripheral olfactory or octavolateralis systems, respectively. It suggests that the relative importance of vision may be higher in *T. maccoyii* as compared to other sensory modalities.

The volumes of six brain areas (optic tectum, corpus cerebellum, olfactory bulb, telencephalon, corpus cerebellum, eminentia granularis, and cristate cerebelli) were previously measured in skipjack tuna *K. pelamis* and other pelagic teleosts (dolphinfish *Coryphaena hippurus*, escolar *Lepidocybium flavobrunneum*, shortbill spearfish *Tetrapturus angustirostris*, striped marlin *Kajikia audax*, and swordfish *Xiphias gladius*), using the idealized ellipsoid method (Lisney and Collin 2006). These values are compared to *T. maccoyii* to understand how different methods affect the quantitative assessment of pelagic teleost brains (Table 3). However, a direct comparison between values is challenging, as the total brain volume is not available in other pelagic teleosts examined (Lisney and Collin 2006). Therefore, when making interspecific comparisons of the relative volume of brain areas to the above pelagic species, the relative volume of different brain areas of *T. maccoyii* is in relation to the sum of these six brain areas (henceforth termed “adjusted brain volume”), but excludes other regions, including the diencephalon and the rest of the medulla oblongata. Due to the use of different quantification methodologies, large discrepancies in brain areas that have large areas not externally visible (e.g., corpus cerebellum) or large ventricular space (e.g., optic tectum) are hypothesized (Ullmann et al. 2011).

**TABLE 3:** Absolute and relative volumes (Vol) for five brain areas in *Thunnus maccoyii* (SBT8) compared to previous investigations on pelagic teleosts by Lisney and Collin (2006). The relative volumes of each brain area are determined by referring them to the sum of the five brain areas. Octavolateralis area refers to the sum of the eminentia granularis and cristae cerebelli.

	Olfactory bulb		Optic tectum		Octavolateralis area		Telencephalon		Corpus cerebellum		Method	Reference
	Vol (mm^3^)	Vol (%)	Vol (mm^3^)	Vol (%)	Vol (mm^3^)	Vol (%)	Vol (mm^3^)	Vol (%)	Vol (mm^3^)	Vol (%)		
*Thunnus maccoyii*	42	2	558	24	122	5	258	11	1299	57	MRI	This study
*Coryphaena hippurus*	107	5	924	42	19	1	959	44	190	9	Idealized/half ellipsoids	Lisney and Collin (2006)
*Lepidocybium flavobrunneum*	40	4	584	61	74	8	159	17	98	10	Idealized/half ellipsoids	Lisney and Collin (2006)
*Katsuwonus pelamis*	32	1	1127	53	62	3	400	19	509	24	Idealized/half ellipsoids	Lisney and Collin (2006)
*Tetrapturus angustirostris*	69	5	874	67	84	6	146	11	139	10	Idealized/half ellipsoids	Lisney and Collin (2006)
*Kajikia audax*	23	3	526	60	14	1	85	10	229	26	Idealized/half ellipsoids	Lisney and Collin (2006)
*Xiphias gladius*	73	7	589	59	14	1	86	9	234	23	Idealized/half ellipsoids	Lisney and Collin (2006)

The relative volume of the optic tectum of *T. maccoyii* accounts for, on average, 28% less than the six other pelagic species (24% vs. 52% of adjusted brain volume) (Table 3) (Lisney and Collin 2006). The discrepancy likely reflects the overestimation inherent in the idealized ellipsoid method (Wagner 2001), which is based solely on external measurements and likely includes midbrain regions not involved in visual processing (Ullmann et al. 2011). Inclusion of adjacent nonvisual structures (i.e., the valvula cerebelli, tectal ventricle, and about one‐third of the corpus cerebelli) increases the absolute optic tectal volume to 1383 mm^3^ (166% increase) and the relative volume to 60%. Based on more accurate segmentation using MRI, the optic tectum comprises 15% of the total brain volume and is not the largest brain region overall, where that distinction belongs to the corpus cerebelli (Table 3). A broader comparison of relative optic tectal volume can be made with a large diversity of diurnal and nocturnal reef‐associated teleosts, whose total brain volumes are available, and the optic tecta are imaged with microCT scans and virtually segmented (Iglesias et al. 2018). The relative volume of the optic tecta is 20 ± 0.4% (mean ± SE, *n* = 78) for the diurnal species and 17 ± 1 % (*n* = 33) for nocturnal species (Iglesias et al. 2018), which is more comparable to the relative optic tecta volume (15%) of *T. maccoyii*. Nocturnal reef‐associated species, despite having larger eyes for improved sensitivity (Schmitz and Wainwright 2011), are suggested to make less neural investment to their optic tecta, due to the reduced detectable visual information in dark conditions; in contrast, diurnal reef‐associated species likely rely on higher visual acuity and color discrimination, which may be subsequently reflected in enlarged optic tecta (Iglesias et al. 2018). Large pelagic teleosts, including *T. maccoyii*, primarily occupy the brightly lit epipelagic zone (Block et al. 1992; Brill et al. 1993; Patterson et al. 2008; Bernal et al. 2017), which is often absent of complex features common to coastal habitats, such as reefs or seagrass beds. Furthermore, these fishes often dive below the thermocline for foraging (Bernal et al. 2017), where the ambient light becomes darker and more monochromatic (Warrant and Locket 2004), and sensitivity over acuity may be prioritized. Accordingly, blue marlin (*Makaira nigricans*), despite occupying clear, bright, tropical habitats (Block et al. 1992), has one of the highest optical sensitivities among teleosts, supported by a large pupil opening, wide photoreceptors, and high spatial summation (Fritsches et al. 2003). However, the visual acuity of *M. nigricans* (8.5 cycles per degree) is much coarser, associated with low ganglion cell density, particularly when compared to diurnal reef‐associated species, such as the blue tuskfish (*Choerodon albigena*) (16 cycles per degree), which lives in a visually complex habitat (Collin and Pettigrew 1989; Fritsches et al. 2003). Hence, if pelagic teleosts have sensitivity‐oriented vision with poor acuity, the relative optic tectum size in large pelagic teleosts may be more comparable to that of the nocturnal reef‐associated teleosts. However, it must be noted that the relative volume of other sensory brain areas has not been measured in the diurnal and nocturnal reef‐associated teleosts (Iglesias et al. 2018), and it is possible that the reduced optic tectum size is still larger than the other sensory brains in nocturnal species.

### 4.2 Cerebellum of *T. maccoyii*

The corpus cerebelli of teleosts is a part of the hindbrain, as it is generally positioned caudal to the midbrain (Butler and Northcutt 1993; Wullimann et al. 1996; Eastman and Lannoo 2004; Cerdá‐Reverter et al. 2008; Lisney and Collin 2006; Ullmann, Cowin, and Collin 2010). However, in *T. maccoyii*, due to its remarkable rostro‐caudal extension, the dorsal component of the corpus cerebelli spans across the roof of the midbrain, from the caudal end of the telencephalon (i.e., forebrain) to the dorsal surface of the medulla oblongata (i.e., hindbrain). The corpus cerebelli of *T. maccoyii* is also foliated, with three distinct horizontal grooves, or sulci, which divide the corpus cerebelli into four parts. The external morphology of the corpus cerebelli of *T. maccoy*ii is shared among tuna species examined to date (Kawamura et al. 1981; Palmieri et al. 2008). Cartilaginous fishes (sharks, rays, skates, and chimaeras) show an incredibly high degree of variation in cerebellar foliation (Yopak et al. 2007; Lisney et al. 2008; Ari 2011; Yopak et al. 2016), which is predicted by body size, brain size, and cerebellum size (Yopak et al. 2019); the highest degree of foliation in this group, which far‐exceeds the foliation of tunas, is found in agile, pelagic species whose well‐foliated corpus cerebelli also expand over the midbrain (Lisney and Collin 2006; Yopak et al. 2007). In contrast, sluggish benthopelagic elasmobranchs (e.g., *Somniosus* spp.) and benthic species possess low levels of cerebellar foliation (Yopak et al. 2007, 2019). In teleosts, such dynamic range in foliation has not been documented; however, species exhibiting any horizontal sulci similarly tend to be large‐bodied, pelagic species (Kawamura et al. 1981; Lisney and Collin 2006; Palmieri et al. 2008; this study). Only one (non‐tuna) pelagic teleost species studied so far, the mahi mahi, *C. hippurus*, exhibits cerebellar folding, although its rostro‐caudal extension is less pronounced than that of tunas or pelagic elasmobranchs (Lisney and Collin 2006; Yopak et al. 2007).

While there is some functional debate, the corpus cerebelli is widely recognized as a center for motor control and motor learning (Bauchot et al. 1977; New 2001; Montgomery et al. 2012). Increased cerebellar surface area, driven by expanded structure volume and/or foliation, is often considered a proxy for greater computational capacity (Sultan 2002). Accordingly, pronounced cerebellar foliation in pelagic teleosts and elasmobranchs aligns with their demanding locomotor performance. Yet, it remains unclear why sluggish benthic/benthopelagic elasmobranchs retain some degree of cerebellar foliation (Yopak et al. 2007, 2019), albeit minimal, whereas many teleosts with comparable or superior locomotor abilities exhibit none (Lisney and Collin 2006; Ito et al. 2007). Interestingly, the corpus cerebelli of billfishes (*K. audax*, *T. angustirostris*, and *X. gladius*) appears to lack foliation or rostral extension, although it shows long caudal extension (Kawamura et al. 1981; Lisney and Collin 2006). This may reflect the immaturity of the specimens examined (Lisney and Collin 2006), with motor control centers not yet fully developed to form sulci. In the blue spotted stingray (*Neotrygon kuhlii*), adult individuals display more pronounced and extensive cerebellar foliation compared to the juveniles (Lisney et al. 2017), and a similar ontogenetic trend is found among sharks (Lisney et al. 2007; Yopak and Frank 2009; Laforest et al., 2020). Alternatively, the reduced foliation of billfishes could correspond to their comparatively slower cruising speed (Block et al. 1992; Domenici et al. 2014; Marras et al. 2015).

The corpus cerebelli of *T. maccoyii* projects into and occupies the medial portion of the midbrain ventricular space. The corpus cerebelli alone occupies 35% of the total brain volume, which is about 2.3 times more than the second largest component, the optic tectum (Table 2). The relative volume of the corpus cerebellum in relation to adjusted brain volume (which includes the olfactory bulbs, telencephalon, optic tectum, corpus cerebellum, eminentia granularis, and cristae cerebelli) was calculated to enable comparisons with other pelagic teleosts investigated by Lisney and Collin (2006). The relative volume of the corpus cerebelli of *T. maccoyii* in this calculation (57%) is much higher compared to that of other pelagic teleosts (23% on average) and *K. pelamis* (24%), which were measured using the idealized ellipsoidal methods (Table 3) (Lisney and Collin 2006). As a large portion of the corpus cerebelli is not visible from the external surface and cannot be measured from dorsal and lateral images in *T. maccoyii*, it is most likely that the relative volumes of the corpus cerebelli in the other pelagic and non‐pelagic teleosts were underestimated by the idealized ellipsoidal method (Lisney and Collin 2006; Edmunds et al. 2016), especially for *K. pelamis* and *C. hippurus*, which have elongated corpus cerebelli, with multiple horizontal sulci (Lisney and Collin 2006). Given that the size of the corpus cerebelli in fishes has been linked to locomotion and motor control (Bauchot et al. 1977; New 2001; Montgomery et al. 2012), it is unsurprising that *T. maccoyii* shows relative enlargement of this brain structure. This region likely supports the processing of large volumes of motor‐related information necessary for precise control of speed, acceleration, and directional changes in an open, 3D pelagic environment (Magnuson 1970; Pavlov et al. 2017; Gleiss et al. 2019). In freshwater habitats, pelagic species similarly show relative enlargement of the cerebellum in comparison to littoral species, likely due to the greater demand in 3D movement coordination (e.g., Edmunds et al. 2016). Additionally, cerebellar expansion may facilitate the locomotive coordination required for synchronized schooling behavior, whether for effective predation or maintaining school cohesion (Partridge et al. 1983).

The valvula cerebelli (410 mm^3^) found in *T. maccoyii*, the third largest brain area quantified, protrudes rostrally into the mesencephalic ventricle and is distinctively divided into medial and lateral lobes. While the rostral protrusion of valvula cerebelli is common, the size or degree of division shows remarkable interspecific differences among teleosts (Yamamoto 2017). For example, the valvula cerebelli of the Japanese ricefish (*Oryzias latipes*) is small without further subdivisions, whereas the valvula cerebelli of elephant nose fish (*Gnathonemus petersii*) is so hypertrophied that it almost completely covers the entire brain (Yamamoto 2017). The exact functional role of valvula cerebelli, a brain region specific to teleosts, remains ambiguous, although it is found to be involved in behaviors like avoidance conditioning, dorsal light response, and processing of non‐motor information (Kaplan et al. 1969; Yanagihara et al. 1993; Chang et al. 2021). Disproportionately large valvula cerebelli of *G. petersii*, an electroreceptive species, suggests its involvement in processing electrosensory information (Yamamoto 2017). However, this hypothesis cannot explain the large and well‐divided valvula cerebelli of large tunas (*T. maccoyii* and Atlantic bluefin tuna, *Thunnus thynnus*) (Palmieri et al. 2008; this study) and goldfish (*Carassius auratus*) (Yamamoto 2017). The local functional circuitry of the valvula cerebelli of mormyrid fishes is found to be similar to that of the mammalian cerebellum (Zhang et al. 2011). Therefore, the enlarged valvula cerebelli of *T. maccoyii* may be associated with locomotion and sensory–motor integration, similar to the corpus cerebelli. Previous work has shown that the common carp (*Cyprinus carpio*), which also has large, well‐divided valvulae cerebelli, sank down onto the bottom after the ablation of the valvula (Ito and Kishida 1978).

### 4.3 Olfactory Bulb of *T. maccoyii*

The olfactory bulbs of *T. maccoyii* occupy the smallest proportion of the measured sensory brain areas, which is consistent with other pelagic teleosts (Table 3) (Lisney and Collin 2006). This is surprising considering the large size of the olfactory nerve, whose diameter is almost as wide as the olfactory bulb. Olfactory receptor neurons (ORNs) project their axons to the olfactory bulb (Caprio 1988; Hara 1992), and an increased number of ORNs (and their afferent axons) has been suggested to drive enlargement of the olfactory bulbs in teleosts (Yamamoto 2017). Additionally, it has been hypothesized that yellowfin tuna *Thunnus albacares* have comparable olfactory sensitivity to the channel catfish, *Ictalurus punctatus*, a species possessing exceptionally sensitive olfactory abilities (Caprio 1977), with the ability to detect amino acids in concentrations as low as 10^−11^ M (Atema et al. 1980).

In teleosts, enlarged olfactory bulbs are typically found in species that hunt in low‐light conditions, where available visual information is limited and smell can provide important cues (Edmunds et al. 2016; Yamamoto 2017). For example, the olfactory bulbs of the kidako moray (*Gymnothorax kidako*), a nocturnal reef‐associated predator, are hypertrophied and larger than the optic tectum (Ito et al. 2007). Similar correlations between enlargement of the olfactory bulbs and habitat type can also be found among elasmobranchs (Yopak et al. 2015). Bathyal (deep‐sea) sharks possess large olfactory bulbs (Yopak et al. 2015), making up more than 30% of the brain in benthopelagic species like *Somniosus microcephalus* and *S. pacificus*, while their optic tecta are greatly reduced (≈2.5%; Yopak et al. 2019). In contrast, reef‐associated sharks, living in visually complex habitats, possess the smallest olfactory bulbs, and, similar to diurnal reef‐associated teleosts (Iglesias et al. 2018), enlarged optic tecta (Yopak and Lisney 2012; Yopak et al. 2015).

Interestingly, some large pelagic elasmobranchs, such as the great white shark (*Carcharodon carcharias*) and blue shark (*Prionace glauca*), combine both large olfactory bulbs and enlarged optic tecta (Lisney and Collin 2006; Yopak and Lisney 2012, Yopak et al. 2015). The hypertrophied olfactory bulbs found in these large pelagic migratory elasmobranchs (Carey and Scharold 1990; Bonfil et al. 2005; Domeier and Nasby‐Lucas 2008) are also observed in highly migratory avians (Bang and Cobb 1968; Rehkamper et al. 1988; Nevitt et al. 2008) and wide‐ranging terrestrial mammals (Gittleman 1991; Safi and Dechmann 2005), and may be linked to enhanced spatial navigation (Jacobs 2012; Yopak et al. 2015). However, given that tunas, especially bluefin tunas, are among the most migratory of all teleosts, capable of transoceanic movements and occupying vast distributional ranges (Patterson et al. 2008, 2018; Fujioka et al. 2018; Block et al. 2019; Faillettaz et al. 2019), their comparatively small olfactory bulbs do not appear to reflect the influence of their highly migratory nature.

Obtaining directional information from chemical cues in an aquatic environment is inherently difficult, as odors themselves are nondirectional; fishes must instead rely on the concentration gradient or temporal intermittency of odor plumes, signals that are often too weak or distorted for localization (Kleerekoper et al. 1975; Atema 1980). In *T. maccoyii*, the olfactory bulb constitutes only 1% of the total brain volume, whereas the optic tectum occupies 15%, suggesting a visually‐mediated over an olfactory‐mediated lifestyle. Yet, as discussed above, the visual system of large pelagic teleosts likely optimizes for sensitivity, with relatively poor acuity, which does not fully justify the investment in optic tectum volume. Moreover, some visually driven, diurnal, reef‐associated teleosts have optic tecta comprising up to 39% of the total brain volume (Iglesias et al. 2018), more than twice that of *T. maccoyii*. Future studies should clarify the apparent mismatch between the well‐developed peripheral olfactory organ and the comparatively small olfactory bulbs in *T. maccoyii*. Counting axon numbers in the olfactory nerve of *T. maccoyii* may provide insight, as deep‐sea grenadiers exhibit ontogenetic increase in olfactory nerve axon counts corresponding to olfactory bulb enlargement and a predicted increased reliance on smell (Lisney et al. 2018). Comparing axon numbers with ORN numbers could further reveal whether “olfactory summation” occurs between the ORNs in this species.

### 4.4 Telencephalon of *T. maccoyii*

The telencephalon comprises 7% of the total brain volume in *T. maccoyii* (Table 2) and 11% of adjusted brain volume, which is smaller than skipjack tuna (19%) (Table 3) (Lisney and Collin 2006). This is likely due to the idealized ellipsoidal method overestimating the volume of the telencephalon, as the lobed nature of the telencephalon in tunas deviates from the shape of the idealized ellipsoid, and as was previously found for the telencephalon of the barramundi (*L. calcarifer*) (Ullmann et al. 2010). Despite its direct connection to the olfactory bulb via the olfactory tract, only a limited area of the telencephalon is involved in receiving and processing olfactory input (Murakami et al. 1983; Finger 1988). Instead, the relative size of this region correlates with ecological parameters, including habitat complexity, social intelligence, and foraging, all functions proposed to reflect higher‐order integrative brain functions (Davis and Kassel 1983; Demski and Northcutt 1996; Huber et al. 1997; Kotrschal et al. 1998; Gonzalez‐Voyer and Kolm 2010; Park and Bell 2010; Edmunds et al. 2016; Axelrod et al. 2021). In cartilaginous fishes, relatively larger telencephalons can be found in reef‐associated or coastal species (Northcutt 1978; Lisney and Collin 2006; Yopak et al. 2007). In comparison, benthopelagic or mesopelagic species living in dim and/or less spatially complex habitats have relatively small telencephalons (Lisney and Collin 2006; Yopak et al. 2007, 2019). Many of the reef‐associated and pelagic sharks and rays that display complex social behaviors, such as coordinated hunting, dominance hierarchies, and courtship activity (Johnsen and Nelson 1973; Gruber and Myrberg 1977; Yano et al. 1999; Ritter and Godknecht 2000), have the largest telencephalons relative to total brain size, with a particular expansion of the dorsal pallium (Lisney and Collin 2006; Yopak et al. 2007; Ari 2011). Similar correlations have been found among teleosts, as species occupying more complex habitats (e.g., reef, littoral) tend to have larger telencephalons compared to those occupying more homogenous habitats (e.g., open water) (Huber et al. 1997; Edmunds et al. 2016), which may explain why the relative volume of telencephalon is smaller compared to other brain areas in *T. maccoyii*. The tropical pelagic teleost mahi mahi (*C. hippurus*) has a much larger relative volume of telencephalon (44% of adjusted brain volume) compared to *T. maccoyii* (11%) or other pelagic teleosts (8%–19%) (Lisney and Collin 2006). As *C. hippurus* displays distinct sexual dimorphism and forms small schools (Palko et al. 1982), relative enlargement of the telencephalon may be attributable to complex social behaviors (Pough et al. 1999). However, tunas also form large cohesive schools and hunt cooperatively with other members to enhance feeding success and gain hydrodynamic benefits (Partridge et al. 1983; Klimley and Holloway 1999). Pacific bluefin tuna *Thunnus orientalis* have also been shown not to fare well when kept isolated in captivity (Dale et al. 2015). Therefore, to better understand the ecological and behavioral factors influencing the size of the telencephalon in large pelagic teleosts, a comparative assessment of the telencephalon across a greater diversity of large pelagic teleosts, including ones that display more solitary behavior (e.g., *X. gladius*), should be investigated.

### 4.5 Diencephalon and Rhombencephalon of *T. maccoyii*

The volume of the inferior lobes (352 mm^3^) of *T. maccoyii* predominates the diencephalon and is the fourth largest among the quantified brain areas, which comprise 9% of the total brain volume (Table 3). As inferior lobe volume has not been measured in other pelagic teleosts, it is difficult to make interspecific comparisons. However, the inferior lobe receives secondary visual input from the optic tectum via the nucleus glomerulosus (Wullimann and Meyer 1990; Butler et al. 1991; Yang et al. 2007) and plays an important role in visual learning and object recognition in teleosts (Calvo et al. 2023). The large absolute and relative volume of the inferior lobes in comparison to other brain areas in *T. maccoyii* may, therefore, be another indicator of the significance of vision. The hypophysis or pituitary gland, which is also part of the diencephalon, has a volume of 22 mm^3^ and comprises 0.6% of the total brain volume in *T. maccoyii*. The pituitary gland of Atlantic bluefin tuna *T. thynnus* shows an immunohistochemical response to gonadotropin‐releasing hormone (Palmieri et al. 2008), which is a key regulator of reproductive function in teleosts (Peter 1983; Okuzawa and Kobayashi 1999; Zohar and Mylonas 2001). However, it is difficult to make any functional correlation between the volume of the hypophysis of *T. maccoyii* and its reproductive function. This would require a comparison of the volume of the hypophysis at different stages of its reproductive maturity.

The combined volume of the eminentia granularis and cristae cerebelli, which receive input from the auditory and the vestibular systems, of *T. maccoyii* is 3% of the total brain volume. This relatively small volume of the octavolateralis area compared to the optic tectum (15%) may support the ecoacoustic constraints hypothesis that teleost species inhabiting environments with high noise levels and weak biological sounds (e.g., open ocean) have poor hearing ability and weak reliance on sound signals (Schellart and Popper 1992; Ladich 2014). Unfortunately, the relative size of the octavolateralis region in comparison to the total brain is mostly unknown in teleosts. It is, however, interesting to note that the combined volume of the eminentia granularis and cristae cerebelli of zebrafish (*Danio rerio*), measured with superior MRI resolution (an isotropic resolution of 10 µm), makes up about 3% of the total brain volume as well (Ullmann et al. 2010). Therefore, to further test the ecoacoustic hypothesis on the CNS, the relative volume of the octavolateralis region of the brain should be investigated in a greater diversity of teleost species that live in quiet stagnant freshwater, as a large portion of freshwater species display anatomical specializations in their peripheral auditory system, which can enhance their hearing ability (Schellart and Popper 1992; Amoser and Ladich 2005, 2014).

## 5 Conclusions

Segmentation of major brain regions of *T. maccoyii* using MRI reveals a relatively large corpus cerebelli, comprising 35% of total brain volume, suggesting a critical role of locomotion, manoeuvrability, and precise control of fin positions in this species. Nonetheless, the optic tectum and associated visual brain structures also comprise a large proportion of total brain volume, particularly in comparison to the olfactory bulb, eminentia granularis, and cristae cerebelli, suggesting an increased reliance on vision in this species. This study demonstrates the advantage of MRI over traditional approximation methods for obtaining accurate brain volumes in teleosts. Specifically, MRI avoids overestimating the size of the optic tectum by excluding ventricle and adjacent nonvisual midbrain structures and prevents underestimating the size of the corpus cerebelli by capturing internal projections not visible externally. Given these approaches, future studies on pelagic teleosts or elasmobranchs should prioritize the use of noninvasive bioimaging methods such as MRI or micro‐computed tomography (Collin et al. 2024), to enable accurate comparisons of sensory and non‐sensory brain regions.

## Author Contributions

**Myoung Hoon Ha**: conceptualization, resources, funding acquisition, investigation, data curation, formal analysis, visualization, writing – original draft, writing – review and editing. **Lucille Chapuis**: conceptualization, methodology, project administration, supervision, validation, writing – review and editing. **Rebecca Glarin**: methodology, data curation. **Bradford Moffat**: methodology, data curation. **David K. Wright**: methodology, data curation. **Travis L. Dutka**: conceptualization, resources, project administration, supervision, writing – review and editing. **Julian Pepperell**: conceptualization, project administration, supervision, writing – review and editing. **Caroline C. Kerr**: conceptualization, project administration, funding acquisition, writing – review and editing. **Kara E. Yopak**: methodology, writing – review and editing. **Shaun P. Collin**: conceptualization, project administration, supervision, funding acquisition, validation, writing – review and editing.

## Funding

This work was financially supported by the Seaworld Research and Rescue Foundation (SWR/4/2023), La Trobe University Graduate Research scholarship and School of Agriculture, Biomedicine, and Environment HDR Student Research Support (to M.H.H.), the Australian Research Council (DP240102532) (to S.P.C.), and the Max Planck Queensland Centre (MPQC) for the Materials Science of Extracellular Matrices (to S.P.C. and C.C.K.).

## Ethics Statement

As this study used animals harvested and donated by recreational fisherman under Victorian Fisheries Authority research permit RP1447, no ethical approval was required.

## Conflicts of Interest

The authors declare no conflicts of interest.

## Supporting information




**Supplementary MoviesS1**: cne70148‐sup‐0001‐MoviesS1.avi

**Supplementary MoviesS2**: cne70148‐sup‐0002‐MoviesS2.avi

**Supplementary MoviesS3**: cne70148‐sup‐0003‐MoviesS3.avi

**Supplementary MoviesS4**: cne70148‐sup‐0004‐MoviesS4.avi

**Supplementary MoviesS5**: cne70148‐sup‐0005‐MoviesS5.avi

**Supplementary Information**: cne70148‐sup‐0006‐SuppMat.docx

## Data Availability

The most relevant data are included in the tables of this article. Additional data are available upon reasonable request.
