# Supplementary material for: Brain Morphology and Quantitative Assessment of Sensory Brain Areas in Southern Bluefin Tuna, Thunnus maccoyii (Scombridae, Teleostei)
Source: J Comp Neurol. 2026 Mar 27;534(4):e70148. doi: 10.1002/cne.70148 (PMC13023358; doi:10.1002/cne.70148)
Supplement: Supplementary file 6 — Supplementary Information: cne70148‐sup‐0006‐SuppMat.docx [file CNE-534-e70148-s002.docx]

**Supplementary Information**

**Brain morphology and quantitative assessment of sensory brain areas in southern bluefin tuna, *Thunnus maccoyii* (Scombridae, Teleostei)**

Myoung Hoon Ha^1*^, Lucille Chapuis^1^, Rebecca Glarin^2^, Bradford Moffat^2^, David Wright^3^, Travis L. Dutka^1^, Julian Pepperell^4^, Caroline C. Kerr^1,5^, Kara E. Yopak^6^, and Shaun P. Collin^1,5^

^1^School of Agriculture, Biomedicine and Environment, La Trobe University, Melbourne, Victoria 3086, Australia

^2^Melbourne Brain Centre Imaging Unit, Dept of Radiology, Medicine, Dentistry and Health Sciences, University of Melbourne, Victoria 3010, Australia

^3^School of Translational Medicine, Monash University, Melbourne, Victoria 3004, Australia

^4^Pepperell Research & Consulting Pty Ltd, 93 Marnie Cres, Doonan, Queensland 4562, Australia

^5^Max Planck Queensland Centre (MPQC) for the Materials Science of Extracellular Matrices, Queensland University of Technology, Kelvin Grove, Queensland 4059, Australia

^6^Department of Biology and Marine Biology, University of North Carolina Wilmington, UNCW Centre for Marine Science, Wilmington, North Carolina 28409, USA

**⁎ Corresponding author**: School of Agriculture, Biomedicine and Environment, La Trobe University, Melbourne, Victoria 3086, Australia. Tel.: + 61 040 778 0646. E-mail address: m.ha@latrobe.edu.au

**Supplementary Information 1**

Dragonfly (Comet Technologies Canada Inc, version 2024) animation of the brain of *T. maccoyii* rotated along the z-axis (yaw). Colour code: cristae cerebelli (dark green); corpus cerebelli (yellow); eminentia granularis (light green); hypophysis (lemon); inferior lobe (orange); medulla (peach); olfactory bulb (light blue); optic chiasma (purple); optic tectum (dark blue); telencephalon (red).

**Supplementary Information 2**

Dragonfly (Comet Technologies Canada Inc, version 2024) animation of the brain of *T. maccoyii* rotated along the x-axis (roll). Colour code: cristae cerebelli (dark green); corpus cerebelli (yellow); eminentia granularis (light green); hypophysis (lemon); inferior lobe (orange); medulla (peach); olfactory bulb (light blue); optic chiasma (purple); olfactory nerve (brown); optic tectum (dark blue); telencephalon (red).

**Supplementary Information 3**

Dragonfly (Comet Technologies Canada Inc, version 2024) animated segmentation of the major structures of the *T. maccoyii* brain in the lateral view. Colour code: cristae cerebelli (dark green); corpus cerebelli (yellow); eminentia granularis (light green); hypophysis (lemon); inferior lobe (orange); medulla (peach); olfactory bulb (light blue); optic chiasma (purple); olfactory nerve (brown); optic tectum (dark blue); telencephalon (red).

**Supplementary Information 4**

Dragonfly (Comet Technologies Canada Inc, version 2024) animated segmentation of the major structures of the *T. maccoyii* brain in the dorsal view. Colour code: cristae cerebelli (dark green); corpus cerebelli (yellow); eminentia granularis (light green); hypophysis (lemon); inferior lobe (orange); medulla (peach); olfactory bulb (light blue); optic chiasma (purple); olfactory nerve (brown); optic tectum (dark blue); telencephalon (red).

**Supplementary Information 5**

Dragonfly (Comet Technologies Canada Inc, version 2024) animation revealing the internal structures of the optic tectum in *T. maccoyii*. The brain (forebrain, midbrain, and cerebellum) of *T. maccoyii* is rotated to show its ventral surface. Then, optic chiasma (purple), diencephalon (pink), corpus cerebelli (yellow), olfactory bulb (light blue), telencephalon (orange), and valvula cerebelli (red) are sequentially removed to reveal the internal structure of the optic tectum (dark blue).
